# Semantically linking and browsing PubMed abstracts with gene ontology

**DOI:** 10.1186/1471-2164-9-S1-S10

**Published:** 2008-03-20

**Authors:** Bhanu C Vanteru, Jahangheer S Shaik, Mohammed Yeasin

**Affiliations:** 1 Electrical and Computer Engineering Department, University of Memphis, Memphis, Tennessee, USA; 2 Bioinformatics Program, University of Memphis, Memphis, Tennessee, USA; 3 Software Testing and Excellence Program, University Of Memphis University of Memphis, Memphis, Tennessee, USA

## Abstract

**Background:**

The technological advances in the past decade have lead to massive progress in the field of biotechnology. The documentation of the progress made exists in the form of research articles. The PubMed is the current most used repository for bio-literature. PubMed consists of about 17 million abstracts as of 2007 that require methods to efficiently retrieve and browse large volume of relevant information. The State-of-the-art technologies such as GOPubmed use simple keyword-based techniques for retrieving abstracts from the PubMed and linking them to the Gene Ontology (GO). This paper changes the paradigm by introducing semantics enabled technique to link the PubMed to the Gene Ontology, called, SEGOPubmed for ontology-based browsing. Latent Semantic Analysis (LSA) framework is used to semantically interface PubMed abstracts to the Gene Ontology.

**Results:**

The Empirical analysis is performed to compare the performance of the SEGOPubmed with the GOPubmed. The analysis is initially performed using a few well-referenced query words. Further, statistical analysis is performed using GO curated dataset as ground truth. The analysis suggests that the SEGOPubmed performs better than the classic GOPubmed as it incorporates semantics.

**Conclusions:**

The LSA technique is applied on the PubMed abstracts obtained based on the user query and the semantic similarity between the query and the abstracts. The analyses using well-referenced keywords show that the proposed semantic-sensitive technique outperformed the string comparison based techniques in associating the relevant abstracts to the GO terms. The SEGOPubmed also extracted the abstracts in which the keywords do not appear in isolation (i.e. they appear in combination with other terms) that could not be retrieved by simple term matching techniques.

## Background

The development of new technologies in the fields of bio-informatics, bio-engineering and functional genomics has lead to the vast amount of research. The advent of these new research fields has lead to an exponential growth of the literature. The PubMed is one of the leading repositories for such growing literature. There are as many as 16 million (and counting) abstracts referenced by the PubMed as of 2006 [1,2]. Finding meaningful abstracts or papers from such a huge database is a great challenge. More than often the classical key-word based search engines yield results that are not meaningful to the query. There is a need of a semantic-sensitive search engine to browse the relevant information from the PubMed.

The search engine used by PubMed is the ‘Entrez’ system [3]. The Entrez system performs the search operation in two steps. In the first step, the Entrez performs the query translation in which it identifies the existence of Medical Subject Headings (MeSH) terms in the query. In the second step, the translated query is compared with words from all the abstracts in the repository based on ‘String Matching’ (term matching) to find the relevant abstracts. The extraction of the relevant abstracts based on string matching can not capture the underlying semantics. If the abstracts were to be retrieved only on the basis of keywords, their synonyms are not used in the search process. For example, Leukemia, blood cancer and bone marrow cancer are synonymous terms. The search in PubMed with the keyword *‘Leukemia’* retrieves only the abstracts containing the word leukemia but not blood cancer/bone marrow cancer. The relevant abstracts are further presented to the user in the order of decreasing PubMed Ids. PubMed Id is the index number for each abstract in the repository. PubMed therefore presents the results in the order of latest to the oldest. This approach causes severe inconvenience to the user to find the relevant abstracts. The bottleneck of this approach is that there is no option to refine the search results from the retrieved abstracts. The user has to manually skim through all the possible abstracts to find the relevant ones since they might be deeply buried inside the retrieved abstracts. There is also a possibility of extracting irrelevant abstracts. For example, the keyword ‘*blood cancer*’ retrieves abstracts that do not have any relevance with that keyword. The closer inspection reveals that all the abstracts in PubMed repository published in the journal ‘*blood cancer research’* are retrieved.

The aforementioned problems are addressed to some extent by the advent of GOPUBMED [4]. The GOPUBMED addresses the problem of refining the search results by introducing the concept of ontology based browsing. The ontology based browsing uses the domain knowledge and taxonomies to hierarchically organize the terms in the given corpus. User query is processed to retrieve relevant abstracts and structure based on the relevance provided by the ontology. For example, the keyword ‘Alzheimer’ is linked to the words ‘brain development’, ‘cell’, ‘memory’ etc. in the Gene Ontology (GO) [5,6]. When the GOPUBMED is searched with the keyword Alzheimer’, the results are displayed categorically based on the relevant keywords ‘brain development’/ ‘cell’ etc. from the GO. It does not however take into account the two main problems viz i) Semantics and ii) Relevance ranking.

The ontology based searching of the large text corpus (for example, PubMed) is an evolving area of research. The research issues addressed in GOPubmed [4] and GO-KDS [7] is closest to the work presented in this paper . The GOPubmed used the concept of ontology-based search into the PubMed [4]. This system organizes the results obtained using PubMed in the order of hierarchical ontology based arrangement. Subsequently, term matching is used to link the abstracts categorically into the GO terms. The process begins with the user submitting the query. The GOPUBMED links to the PubMed via the e-utilities provided by the Entrez System to retrieve the relevant abstracts. The retrieved abstracts are categorized based on the ontology terms using a basic term matching algorithm. The abstracts thus may be browsed categorically based on the ontology terms. This process yields information similar to the information obtained from the GO. The GO already provides the information of linking abstracts to the GO terms [5]. The curators of the Gene Ontology Consortium manually annotate this information. In this regard, GOPUBMED provides redundant information already available from GO.

The other method GO-KDS [7] uses a machine learning approach to address the mentioned objectives in section I. The well-annotated abstracts linked to the GO terms are obtained from various sources such as SwissProt, GenBank, and FlyBase etc. This annotated set of 26500 abstracts published prior to 2001 are linked to 3700 GO terms is used to train the support vector machine (SVM) system. The trained SVM system is validated using the abstracts obtained in 2001. The system performed with an accuracy of 70.5 %. The linking of 26500 abstracts is further generalized to 12 million abstracts (in 2001). It was claimed that the 70.5% accuracy obtained on the training set is acceptable and generalizes this result to 12 million abstracts. The 26500 abstracts considered for training may only address a small proportion of diversification posed by 12 million abstracts. Hence, this procedure suffers severe scaling problems.

The other related works include (but not limited to), ALIBABA [8] which represents the relations among cells, diseases, drugs, proteins, species and tissues as a inter-connected graph extracted from PubMed. Another work, called, PubFinder [9], requests the abstracts of interest from the user. The abstracts are next scanned to find the list of words, which are indicative of discrimination between the abstracts. These words are used to find the relevant abstracts from the PubMed. The MedMiner [10] is another related work in which the user is asked for list of gene names or processes. These words are used as the query terms for GeneCards, which is similar to PubMed. The underlying processes and techniques have not been clearly understood from this paper [10]. A natural language processing (NLP) based approach to find the relations among the genes, proteins and drugs is incorporated into an online application called Chilibot [11]. Another application which is specific towards finding the relations between two proteins is [12]. This application inspects the frequency of the terms in the abstracts for extract the relevant abstracts related to the proteins[12]. This application is also based on direct term matching concept of query and keywords from abstracts. The semantics of the query and the abstracts are not addressed by any of these systems. This will be the main emphasis of this paper.

This paper presents the concept of Semantics Enabled linking of GO with PubMed, called, SEGOPubmed to address the aforementioned problems. The SEGOPubmed adapts the concept of latent semantic analysis (LSA) [13] for linking the PubMed abstracts to the Gene Ontology. The basic idea behind LSA is to map both the documents and the query vector into semantic space before comparison. This process addresses the problem of synonymy by projecting the vectors into low-dimensional space in retrieving abstracts. The comparison between the query and database entries is performed using similarity measure. The cosine similarity measure is found to be well suited for this application. The scores obtained using the similarity measure may be utilized for relevance ranking of the abstracts.

## Results

### Analysis using well referenced keywords

This section presents the performance analysis of SEGOPubmed. To assess the performance of the proposed method, the comparison of the SEGOPubmed is made with the earlier proposed methods for ontology based literature search, namely GOPubmed. The analysis is performed using a few well-referenced query words such as ‘Levimisole Inhibitor’ and ‘rab 5’. The PubMed is enquired using these keywords for extracting the abstracts. The retrieved abstracts are organized semantically using GO terms as tags.

The word ‘Levimisole Inhibitor’ retrieves abstracts related to the enzymes that inhibit the affect of the drug Levimisole. The search using this keyword retrieves 136 abstracts that are further organized semantically using the GO terms. In this paper, three GO terms viz. ‘cell growth’, ‘collagen’, and ‘pathogenesis’ are used to evaluate the performance of SEGOPubmed. The keyword ‘cell growth’ is present in 2 out of 136 abstracts, which is evident from GOPUBMED. There is a possibility of other abstracts that might be related to cell growth but do not contain that keyword. For example, the abstract, PMID: 8267680 deals with the affect of alkaline phosphatase in drug resistant tumor cells. This study analyzes the affect of alkaline phosphatase on cell growth that semantically may be extracted using SEGOPubmed. The analysis using the SEGOPubmed extracted 5 abstracts that were rendered highly ranked to be semantically related to ‘cell growth’. The other abstracts include PMID: 11139434 which talks about anticancer activity of Levimisole, PMID: 15601852 which addresses differentiation (division of cells) cascade of growth plate chondrocytes. The other two abstracts extracted by SGP are PMID: 9213309 and PMID: 9599668 which contain the word ‘cell growth’. The abstracts are relevance ranked in the order PMIDs: 8267680, 9599668, 11139434, 15601852 and 9213309.

The next GO term used is ‘collagen’, which is tensile rich protein of connective tissue. There are 6 abstracts retrieved by the SGP, 5 of which are also retrieved by the GOPUBMED. The abstract PMID: 3936345 relevance ranked #5 by the SEGOPubmed is not retrieved by term matching techniques. This abstract talks about the inflammatory responses in the collagen-induced arthritis models. Although it has the word collagen, it does not exist as a separate word and hence not retrieved by GOPUBMED. The relevance ranking for the word collagen is in the order PMIDs: 9284952, 10647622, 15601852, 2725422, 3936345 and 10983877.

The other query term used in this study is ‘Rab 5’, which yielded 623 abstracts. Rab 5 is a protein that controls the fusion between early endosomes and endocytic vesicles. The GO term ‘pathogenesis’ is used to find abstracts that are semantically related to the keyword. The analysis using SGP resulted in 5 abstracts whose PMIDs are 16113213, 15367862, 15304337, 1554866, and 11785977 in the decreasing order of their relevance. The direct term matching techniques for the above scenario would result in three abstracts which do not include the abstracts 1 and 5 provided by SGP. The close examination of these abstracts reveals that ‘pathogenesis’ does not occur as a single word in one of the abstracts (PMID: 16113213) and the other abstract (PMID: 11785977) semantically addresses the issues of ‘pathogenesis’.

The above empirical analyses reveal that SEGOPubmed incorporates semantics into the ontology based searching of the PubMed. The organization of abstracts according to the relevance would greatly enhance the search experience of the user. Further, the thresholding technique would provide only relevant abstracts to the user.

### Statistical evaluation of SEGOPubmed using GO curated term associations

This section outlines the empirical analysis of the proposed SEGOPubmed from GO curated term associations. The construction of ground truth using GO is shown first.

#### Construction of ground truth using GO

The GO is a consortium that aims to describe the genes and gene products of any organism by providing a controlled vocabulary. The GO extracts the genes/gene products by manually reading the PubMed abstracts and associates them with the vocabulary. This process results in the association of the GO terms with the PubMed abstracts. These series of associations may be downloaded and used as ground truth to evaluate the performance of SEGOPubmed.

#### Empirical evaluation

The statistically evaluation of the performance of SEGOPubmed is performed using the ground truth constructed as described in the previous section. The ground truth consists of 491 PubMed abstracts associated with the 60 GO terms with Ids GO:0000001 to GO:0000070 (some of the terms with Ids such as GO:0000069, GO:0000065 are missing making the count to 60). The SEGOPubmed is queried with the GO terms and the significant abstracts are retrieved by applying R-test and are compared with the ground truth. The number of true positives and false positives among the retrieved abstracts are recorded to build the receiver operating characteristic (ROC) curves. In signal detection theory, a ROC curve is a plot of true positive fraction Vs. false positive fraction. The ROC curves are one of the ways to analyse the cost benefit ratio. The problem at hand is a binary classifier where the abstract is either associated to GO term or not. There are four possible alternatives that may be obtained from the classifier viz. true positives (TP), false positives (FP), true negatives (TN) and false negatives (FN). The TP is the number of truly associated abstracts among the retrieved abstracts. The FP is the number of un-associated abstracts among the retrieved abstracts. On the contrary, TN is the number of truly un-associated abstracts among the rejected abstracts by the SEGOPubmed. FN is the number of truly associated abstracts rejected by the SEGOPubmed. The plot of TPF Vs. FPF hence, enables the comparison of performance of various classifiers employed in the study.

The detailed steps of the performed evaluation may be listed as:

1. Construct the Ground truth by downloading GO terms and their associated abstracts.

2. Query the SEGOPubmed using the GO terms as the query words.

3. Find the significant abstracts using the R-test (see methods).

4. Compare the retrieved abstracts with the ground truth.

5. Calculate the true positive fraction and false positive fraction.

6. Construct the ROC curve

**Figure 1:**
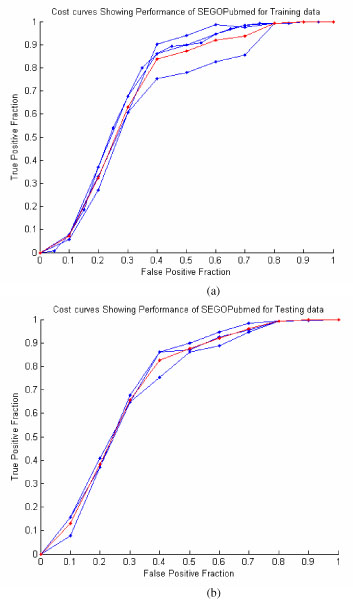
**ROC curves showing the performance of SEGOPubmed** a) training data and b) test data

The validation for both testing and training is done using three GO terms i) ribosomal chaperone activity, ii) transition metal ion transport and iii) autophagic vacuole fusion in step 5. The red curve indicates the average of the 3 roc curves. The Fig. 1 (a) shows the cost curve of the performance of SEGOPubmed for the training dataset. As shown in Fig.1 (a), the SEGOPubmed recorded very small FPF and very large TPF. This indicates that the model performed well for the training data. Fig. 1(b) shows the cost curve for the test data. Fig. 1(b) shows the similar performance as seen for the training data and hence can be used to classify the test documents to the GO terms.

The cost curves plotted show the performance of SEGOPubmed for only 3 GO terms. For a complete investigation of the performance for all the 60 GO terms considered, TPF and FPF values at the threshold given by the R-test are mentioned in tables 1 and 2.

**Table 1:** TPF and FPF values for all the 60 GO terms in the training data

**GO term**	**Positives**	**TP**	**FP**	**TPF**	**FPF**
mitochondrion inheritance	10	6	4	0.6	0.005442177
mitochondrial genome maintenance	29	9	6	0.6	0.008219178
reproduction	9	7	2	0.777777778	0.002717391
biological process unknown	13	6	7	0.461538462	0.009562842
ribosomal chaperone activity	10	2	8	0.2	0.010884354
high affinity zinc uptake transporter activity	20	8	7	0.533333333	0.009589041
low-affinity zinc ion transporter activity	7	3	4	0.428571429	0.005420054
thioredoxin	19	0	15	0	0.020547945
alpha-1,6-mannosyltransferase activity	10	3	7	0.3	0.00952381
trans-hexaprenyltranstransferase activity	13	0	13	0	0.017759563
vacuole inheritance	10	7	3	0.7	0.004081633
single strand break repair	18	4	11	0.266666667	0.015068493
single-stranded DNA specific	7	5	2	0.714285714	0.002710027
phosphopyruvate hydratase complex	10	0	10	0	0.013605442
lactase activity	11	8	3	0.727272727	0.004087193
alpha-glucoside transport	17	3	12	0.2	0.016438356
regulation of DNA recombination	30	13	2	0.866666667	0.002739726
regulation of mitotic recombination	34	9	6	0.6	0.008219178
negative regulation of recombination	19	6	9	0.4	0.012328767
mitotic spindle elongation	10	2	8	0.2	0.010884354
maltose metabolism	7	4	3	0.571428571	0.004065041
maltose biosynthesis	9	0	9	0	0.012228261
maltose catabolism	16	13	2	0.866666667	0.002739726
alpha-1,2-mannosyltransferase activity	17	0	15	0	0.020547945
ribosomal large subunit assembly	22	4	11	0.266666667	0.015068493
ribosomal small subunit assembly	10	1	9	0.1	0.012244898
mannosyltransferase activity	26	9	6	0.6	0.008219178
mannosylphosphate transferase activity	14	5	9	0.357142857	0.012311902
cell wall mannoprotein biosynthesis	12	6	6	0.5	0.008185539
alpha-1,3-mannosyltransferase activity	7	4	3	0.571428571	0.004065041
adenine deaminase activity	23	9	6	0.6	0.008219178
acyl binding	21	7	8	0.466666667	0.010958904
acyl carrier activity	7	6	1	0.857142857	0.001355014
very-long-chain fatty acid metabolism	24	4	11	0.266666667	0.015068493
plasma membrane long-chain	12	7	5	0.583333333	0.006821282
low affinity iron ion transport	7	0	7	0	0.009485095
transition metal ion transport	32	7	8	0.466666667	0.010958904
protein targeting to Golgi	17	2	13	0.133333333	0.017808219
ascorbate stabilization	17	0	15	0	0.020547945
autophagic vacuole formation	21	6	9	0.4	0.012328767
autophagic vacuole fusion	14	3	11	0.214285714	0.01504788
Rieske iron-sulfur protein	17	1	14	0.066666667	0.019178082
peptidyltransferase activity	31	11	4	0.733333333	0.005479452
tRNA binding	14	2	12	0.142857143	0.016415869
urea cycle	9	0	9	0	0.012228261
urea cycle intermediate metabolism	31	6	9	0.4	0.012328767
citrulline metabolism	10	0	10	0	0.013605442
argininosuccinate metabolism	11	0	11	0	0.014986376
ribosome export from nucleus	11	9	2	0.818181818	0.002724796
ribosomal large subunit export	17	13	2	0.866666667	0.002739726
ribosomal small subunit export	9	1	8	0.111111111	0.010869565
citrulline metabolism	10	0	10	0	0.013605442
argininosuccinate metabolism	11	0	11	0	0.014986376
protein import into nucleus, docking	13	5	8	0.384615385	0.010928962
protein import into nucleus, translocation	18	0	15	0	0.020547945
protein import into nucleus, substrate release	9	6	3	0.666666667	0.004076087
acyl-CoA binding	8	3	5	0.375	0.006784261
L-ornithine transporter activity	7	5	2	0.714285714	0.002710027
mitochondrial ornithine transport	29	7	8	0.466666667	0.010958904

**Table 2:** TPF and FPF values for all the 60 GO terms in the training data

**GO term**	**Positives**	**TP**	**FP**	**TPF**	**FPF**
mitochondrion inheritance	4	2	2	0.5	0.006802721
mitochondrial genome maintenance	12	5	7	0.416666667	0.024475524
reproduction	3	3	0	1	0
biological process unknown	5	3	2	0.6	0.006825939
ribosomal chaperone activity	4	2	2	0.5	0.006802721
high affinity zinc uptake transporter activity	8	8	0	1	0
low-affinity zinc ion transporter activity	3	2	1	0.666666667	0.003389831
thioredoxin	8	0	8	0	0.027586207
alpha-1,6-mannosyltransferase activity	3	0	3	0	0.010169492
trans-hexaprenyltranstransferase activity	5	0	5	0	0.017064846
vacuole inheritance	3	3	0	1	0
single strand break repair	7	3	4	0.428571429	0.013745704
single-stranded DNA specific	3	2	1	0.666666667	0.003389831
phosphopyruvate hydratase complex	3	1	2	0.333333333	0.006779661
lactase activity	4	3	1	0.75	0.003401361
alpha-glucoside transport	6	1	5	0.166666667	0.017123288
regulation of DNA recombination	12	7	5	0.583333333	0.017482517
regulation of mitotic recombination	14	3	11	0.214285714	0.038732394
negative regulation of recombinations	7	1	6	0.142857143	0.020618557
mitotic spindle elongation	3	1	2	0.333333333	0.006779661
maltose metabolism	3	0	3	0	0.010169492
maltose biosynthesis	3	2	1	0.666666667	0.003389831
maltose catabolism	6	5	1	0.833333333	0.003424658
alpha-1,2-mannosyltransferase activity	6	0	6	0	0.020547945
ribosomal large subunit assembly	9	3	6	0.333333333	0.020761246
ribosomal small subunit assembly	4	0	4	0	0.013605442
mannosyltransferase activity	11	7	4	0.636363636	0.013937282
mannosylphosphate transferase activity	5	2	3	0.4	0.010238908
cell wall mannoprotein biosynthesis	4	2	2	0.5	0.006802721
alpha-1,3-mannosyltransferase activity	3	3	0	1	0
adenine deaminase activity	9	8	1	0.888888889	0.003460208
acyl binding	8	4	4	0.5	0.013793103
acyl carrier activity	3	2	1	0.666666667	0.003389831
very-long-chain fatty acid metabolism	10	1	9	0.1	0.03125
plasma membrane long-chain	4	3	1	0.75	0.003401361
low affinity iron ion transport	3	0	3	0	0.010169492
transition metal ion transport	13	5	8	0.384615385	0.028070175
protein targeting to Golgi	6	2	4	0.333333333	0.01369863
ascorbate stabilization	6	0	6	0	0.020547945
autophagic vacuole formation	8	3	5	0.375	0.017241379
autophagic vacuole fusion	5	2	3	0.4	0.010238908
Rieske iron-sulfur protein	6	2	4	0.333333333	0.01369863
peptidyltransferase activity	13	5	8	0.384615385	0.028070175
tRNA binding	6	1	5	0.166666667	0.017123288
urea cycle	3	2	1	0.666666667	0.003389831
urea cycle intermediate metabolism	13	1	12	0.076923077	0.042105263
citrulline metabolism	4	0	4	0	0.013605442
argininosuccinate metabolism	4	0	4	0	0.013605442
ribosome export from nucleus	4	4	0	1	0
ribosomal large subunit export from nucleus	6	3	3	0.5	0.010273973
ribosomal small subunit export from nucleus	3	2	1	0.666666667	0.003389831
protein import into nucleus, docking	5	2	3	0.4	0.010238908
protein import into nucleus, translocation	7	1	6	0.142857143	0.020618557
protein import into nucleus, substrate release	3	3	0	1	0
acyl-CoA binding	3	2	1	0.666666667	0.003389831
L-ornithine transporter activity	3	2	1	0.666666667	0.003389831
mitochondrial ornithine transport	12	4	8	0.333333333	0.027972028

## Conclusions

This paper opens a new paradigm to semantic-sensitive ontology based browsing and linking of large corpus (i.e., PubMed) to ontologies (for example, GO). The LSA technique is applied on the PubMed abstracts obtained based on the user query and the semantic similarity between the query and the abstracts. The analysis using well-referenced keywords show that the proposed semantic-sensitive technique outperformed the string comparison based techniques in associating the relevant abstracts to the GO terms. The SEGOPubmed also extracted the abstracts in which the keywords do not appear in isolation (i.e. they appear in combination with other terms) that could not be retrieved by simple term matching techniques. The present study is limited to only a few well-referenced keywords. A comprehensive and evaluation based on semantic-space similarity of the SEGOPubmed is currently under investigation. The present technique also does not incorporate the concept of polysemy in linking the abstracts to the GO terms. This feature may be introduced into ontology-based search by using Probabilistic Latent Semantic Analysis (PLSA) that is a probabilistic variant of the LSA.

## Methods

**Figure 2:**
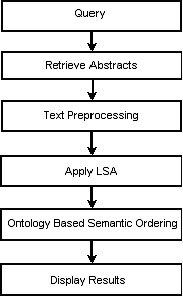
Schematic diagram of the proposed SEGOPubmed

The PubMed is one of the highly used repository for biomedical literature [1]. The results from the PubMed query are arranged in the order of entry of the abstracts into the repository. The order of arrangement intended by most of the users is the relevance of the abstracts to the query. The non-availability of such a feature forces the user to skim through the abstracts to obtain the relevant abstracts. The ontology-based search is hence a most relevant alternative. The LSA is incorporated into this framework to find the semantically meaningful and relevant abstracts. The abstracts are ordered based on relevance and ontology based terms. The main building blocks of the SEGOPubmed are shown in the Fig. 2. As shown in Fig. 2, the user first inputs the query. The relevant abstracts are obtained from the PubMed. The text processing is performed on these abstracts and term frequency (TF) and inverse document frequency (IDF) are obtained. The LSA is performed using the TF and IDF. This process incorporates the semantics into the retrieved document space. Next the GO terms are mapped into the semantic space and semantically related abstracts are retrieved and displayed based on relevance tagged to each of the GO terms.

### Creation of corpus

The process begins by collecting text (for example, PubMed abstract) into a corpus. First, text pre-processing is performed to extract the meaningful words from the abstracts. This is performed by i) eliminating the stop words, ii) stemming the words to their root words and iii) forming the dictionary from all the stemmed words. The irrelevant words are eliminated following the list of words provided by [14]. These are the words that do not offer any significant improvement in the semantics or the search retrieval and also introduce noise in the corpus. A porter-stemming algorithm is used to stem the words in the document to their root words [15].

A matrix is created from the corpus, having one row for each unique word (for example, GO terms) in the corpus and one column for each document (PubMed abstract). Weightings and normalizations are often applied to the data matrix that take into account the frequency of key word *i* (k_i_) in the document *j* (d_j_) and the frequency of k_i_ across all documents, such that distinctive words that appear infrequently are given the most weight. The cells of the matrix consist of weighted term-frequency (T-F) and inverse document frequency (IDF) matrix described in the previous section. Since many words do not appear in any given document, the matrix is often sparse.

The term-frequency (TF) matrix is constructed as proposed Landauer et al [16]. The Inverse Document Frequency (IDF) matrix is constructed using the Eq. 1.

(1) $$idf=\log\left(\frac{\left|D\right|}{\left|\left(d_{i}⊃t_{i}\right)\right|}\right).$$

Here, |D| is the total number of documents in the corpus and $\left|\left(d_{i}⊃t_{i}\right)\right|$ is the total number of documents where the term $t_{i}$ appears [17].

The TF and IDF are next multiplied to form a TF-IDF matrix. The next step as shown in Fig. 2 is to apply LSA using the TF and IDF matrices generated in the text pre-processing step.

### Latent semantic analysis (LSA)

The TF-IDF data matrix (A) is first normalized across the rows by dividing frequency of the word in each document by the highest frequency of that word in all the documents. The LSA transforms the high dimensional TF-IDF data matrix (A) into a low-dimensional latent space through a mathematical procedure known as singular value decomposition (SVD) [18]. SVD is a technique that creates an approximation of the original word by document matrix. After SVD, the original matrix is equal to the product of three matrices, word by latent concept, latent concept by latent concept and latent concept by document. The size of each latent concept (singular value) corresponds to the amount of variance captured by corresponding Eigen vector. Because the singular values are ordered in decreasing size, it is possible to remove the smaller dimensions and still account for most of the variance. The approximation to the original matrix is optimal, in the least squares sense, for any number of dimensions one would choose. In addition, the removal of smaller dimensions introduces linear dependencies between words that are distinct only in dimensions that account for the least variance. Consequently, two words that were distant in the original vector space can be near in the compressed space, causing the inductive semantic space and knowledge acquisition effects reported in the [19].

The SVD is a matrix factorization technique that decomposes the TF-IDF matrix into three different matrices as shown in Eq. 2.

(2) $$A=USV^{T}.$$

The first s Eigen vectors are considered for mapping the high-dimensional TF-IDF data matrix (A) to the low-dimensional space as shown in the Eq. 3.

(3) $$A\approx U_{S}S_{S}V_{S}^{T}.$$

Besides facilitating the dimensionality reduction, the semantic relations are also incorporated in the reduced dimensional space. The GO terms are used as the query vector (q_0_) to inquire the Eigen mapped documents. Since the comparison needs to be performed in the same space, the query expansion is performed by mapping the query vector to the Eigen space as shown in Eq. 4.

(4) $$q=q_{0}^{T}U_{s}\sum_{s}^{-1}.$$

The similarity of each expanded query is found with respect to the document vectors, which are also mapped to Eigen space. This enables the comparisons of the query and the documents based on semantics. A cosine similarity is used as a measure of similarity as proposed in [18]

(5) $$\cos\theta_{j}=\frac{d_{j}^{T}q}{{\left\|d_{j}\right\|}_{2}{\left\|q\right\|}_{2}}.$$

The breakthrough provided by LSA is a solution to the synonymy problem, i.e., the problem that multiple words can express the same meaning. In the basic vector space model, distinct words with the same meaning are kept distinct, but LSA gives them equivalent or near equivalent meanings. LSA's solution to the synonymy problem made it attractive to a variety of researchers outside of information retrieval. The geometrical interpretation provided in [20] gives an insight into the underlying principles of LSA. In general, there are many parameters (for example selecting threshold) that need to be determined for any semantic space. Most often performance of semantic is evaluated by human experts.

### Automated thresholding to extract semantically relevant documents

An R-test is employed to extract semantically relevant documents for a query[21]. The following procedure describes the R-test

**Figure 3:**
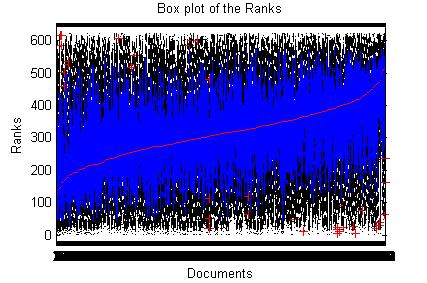
Box plots of the possible ranks for one query

i) Randomly select the terms from the term document matrix.

ii) Find the relevance (ranking using similarity measure or other ways) of the query with the documents based on the GO and PubMed term document space.

iii) Repeat the steps 1 and 2 for 25 iterations as proposed in [21].

iv) Arrange the document ranks based on the median rank (*r*) as shown in the Fig. 3.

**Figure 4:**
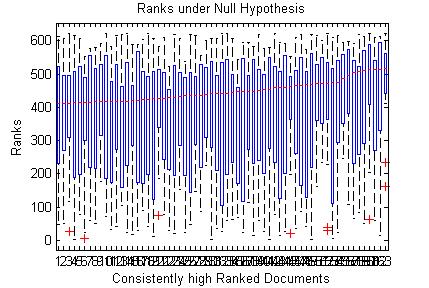
Box plots of the documents under null hypothesis for one query

v) Consider the consistently high ranked documents under null hypothesis. These ranks will follow a uniform distribution as shown in the Fig. 4.

vi) For each document, find the p-value (p) as given by the Eq. 6

(6) $$p=p(r_{i}/g\in UI).$$

The documents that are considered significant using p-value are considered relevant and displayed in the order of relevance. This process is repeated for all the GO terms used as query vectors. The user can now skim through the results using ontology terms based on relevance. Please note that only relevant abstracts are displayed because of the thresholding process.

## Competing interests

The authors declare that they have no competing interests.

## Authors' contributions

Bhanu Chander Vanteru carried out the following tasks i) curated abstracts from PubMed repository, ii) Parsed the XML files, iii) performed preprocessing steps such as stemming, stop word removal, iv) constructed TF, IDF matrices.

Jahangheer Shareef Shaik carried out the following tasks i) performed latent semantic analysis with Vanteru, ii) performed R-test, iii) performed comparison with literature along with Vanteru and iv) participated in manuscript writing along with Vanteru.

Mohammed Yeasin carried out the following tasks: i) defining the problem (ii) supervision of the whole work from inception to completion iii) revised the manuscript and provided critical comments to improve the intellectual merit of the contribution.

All the authors read and approved the final manuscript.
